# Signalling architectures can prevent cancer evolution

**DOI:** 10.1038/s41598-020-57494-w

**Published:** 2020-01-27

**Authors:** Leonardo Oña, Michael Lachmann

**Affiliations:** 1 Department of Ecology, School of Biology/Chemistry, Osnabrück University, Osnabrück, Germany; 2 Santa Fe Institute, Santa Fe, NM 87501 USA

**Keywords:** Cancer prevention, Evolutionary theory

## Abstract

Cooperation between cells in multicellular organisms is preserved by an active regulation of growth through the control of cell division. Molecular signals used by cells for tissue growth are usually present during developmental stages, angiogenesis, wound healing and other processes. In this context, the use of molecular signals triggering cell division is a puzzle, because any molecule inducing and aiding growth can be exploited by a cancer cell, disrupting cellular cooperation. A significant difference is that normal cells in a multicellular organism have evolved in competition between high-level organisms to be altruistic, being able to send signals even if it is to their detriment. Conversely, cancer cells evolve their abuse over the cancer’s lifespan by out-competing their neighbours. A successful mutation leading to cancer must evolve to be adaptive, enabling a cancer cell to send a signal that results in higher chances to be selected. Using a mathematical model of such molecular signalling mechanism, this paper argues that a signal mechanism would be effective against abuse by cancer if it affects the cell that generates the signal as well as neighbouring cells that would receive a benefit without any cost, resulting in a selective disadvantage for a cancer signalling cell. We find that such molecular signalling mechanisms normally operate in cells as exemplified by growth factors. In scenarios of global and local competition between cells, we calculate how this process affects the fixation probability of a mutant cell generating such a signal, and find that this process can play a key role in limiting the emergence of cancer.

## Introduction

**Figure 1:**
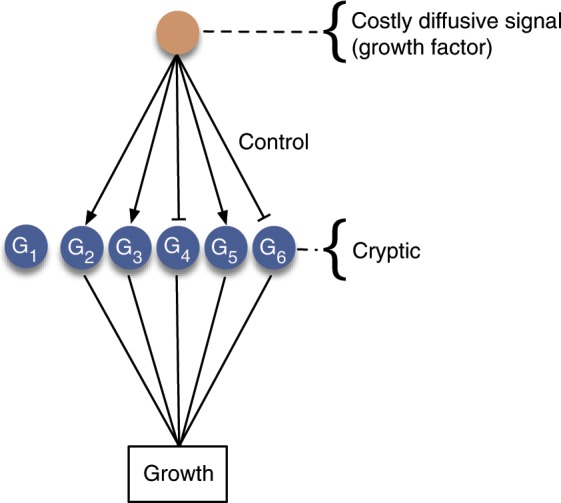
Mechanisms preventing cancer formation. A costly diffusive signal, for example, a growth factor, can regulate the expression of several genes. To generate an effect equivalent to the action of the diffusive signal a particular set of genes have to exhibit a particular expression pattern. As suggested by the multistage theory, this involves a hard-to-evolve, cryptic solution where several mutations are required to produce the particular pattern of gene expression involving a set of stages where the expression of each gene G1, G2 …, G6 should be modified, and that will trigger uncontrolled cell division.

The prevention of cancer has been a major challenge to multicellularity since its origin. It is primarily critical in animals, where cells are mobile and can spread over the multicellular body. The many mechanisms that evolved to counter this proliferation show that cancer puts a substantial evolutionary pressure on animals. Such mechanisms include the immune system and associated tumour antigens such as p53, processes resulting in the limitation of cell growth such as shortening of teleomeres, and tumour suppressor genes such as APC^1–3^. Furthermore, it has been claimed that tissue architecture, and especially of tissues undergoing controlled cell growth has been shaped to prevent cancer^4^. Finally, the multistage theory of cancer states that a tumour requires several mutations to become malignant, showing that organisms in which only few mutations can lead to cancer are selected against^5^ (Fig. 1). For an overview of such mechanisms see^6^.

**Figure 2:**
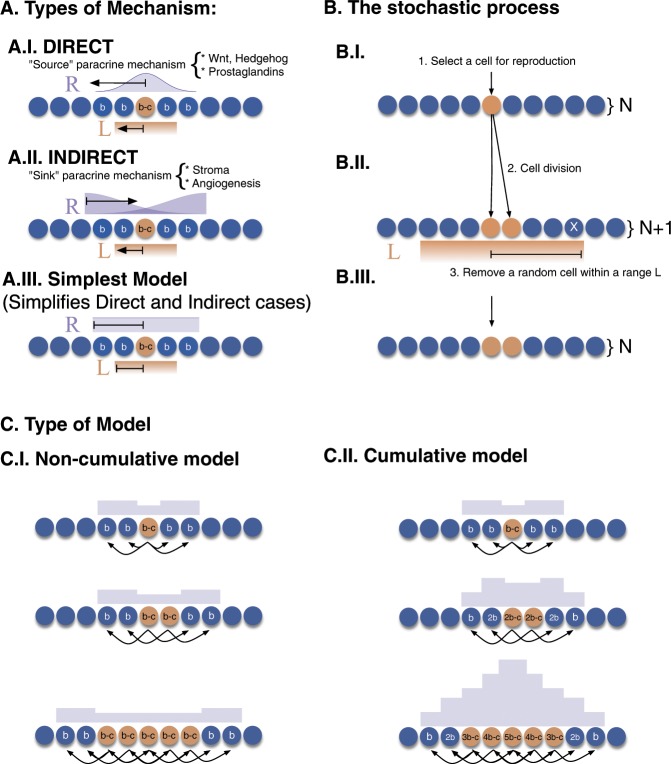
(**A**) Mechanisms where a mutation affects the fitness of cells in the vicinity. In the direct mechanism (A.I.) a mutated cell (red) generates a molecular signal (source) that affects a region around it. In the indirect mechanism, the signal produced by the mutated cell generates a signal in response also affecting a region around it (sink). The simplest model (A.III.) simplifies both mechanisms. The region affected by the signal has a radius *R*. Cells affected by the signal get a benefit *b*, and cells producing the signal pay a cost *c*. The replacement of cells in the stochastic process is done within a region *L*, which can be larger or smaller than *R*. (**B**) Steps of the stochastic process. (**C**) Models of benefit accumulation. In the “Non-cumulative model” (C.I.), the amount of benefit that each of the cells affected by the signal obtains is the same, and it is independent of the number of mutated cells. In the “Cumulative model” (C.II.), the amount of benefit that each of the cells affected by the signal obtains depends on the number of mutated cells. In both examples from C.I and C.II. we have assumed *R* = 2.

Hanahan and Weinberg^7^ have proposed that cancer comprise six biological capabilities acquired during the multistep development of tumours: (1) self-sufficiency in growth signals, (2) insensitivity to growth-inhibitory (antigrowth) signals, (3) evasion of programmed cell death (apoptosis), (4) limitless replicative potential, (5) sustained angiogenesis, and (6) tissue invasion and metastasis. It is interesting to note that at least three (1, 2, and 5) are tightly linked to intercellular signals. (3 and 6 have also been shown to be linked to growth factors^8^). Each of these alterations has to emerge, for example through a mutation, and rise to a sufficient frequency within the tumour – each of these properties evolves in the cancer and needs to provide a selective benefit to the cells that carry it. Several alterations can generate a pre-cancer state, but cannot take hold because the mutation eventually disappeared through negative selection, drift, or because one of the mechanisms mentioned above successfully eliminated it. We show how the signalling system of the organism can be set up so that a mutant that signals for growth (direct mechanism) or for requesting resources (indirect mechanism), will be selected against among its neighbouring cells (Fig. 2). This process can operate against an emerging mutant both in a population of non-proliferating cells or within a tumour.

Growth factors provide one example of signalling molecules that trigger cell growth. They are released into the intercellular space, and can then cause a proliferation not only in the cell that produced it, but also to other cells in the vicinity. If the signal is costly to the producer, these other cells could then out-compete the signaller genotype. The selection process triggered by growth factor production has been previously described^9,10^. In a study exploring the role of the growth factors on tumour heterogeneity, the authors were able to measure the production cost of the growth factor IGF^11^. It has been previously suggested that some signals, in particular neurotransmitters^12^ and signals of quality between cells in the body^13^ would be costly. Here, we focus on signals that would provide a benefit to cancer, and study the implications of cost and range of such signals.

In this paper, we propose a new mechanism for cancer avoidance. We claim that signalling between cells within multicellular organisms exhibit architectures that can result in a reduction in the probability of developing cancer. In particular, we show that such protection against cancer will be present if signals that could benefit cancer cells are costly and have a wide area of effect. We will start by describing our model, and the various versions tested - cumulative benefit to receiving the signal from multiple cells or non-cumulative, one and two dimensional compartments, and different ranges of diffusion and competition. We present an analysis of the simpler models, and simulation results for the more complex ones, showing that these mostly agree. Finally, in the discussion, we provide more empirical evidence for our assumptions, make predictions that follow from our results, and discuss the relationship between the cost as presented in our model with costly signalling theory.

## Model

We studied the effect of a mutation resulting in an increase in the expression of a costly diffusive molecule (i.e., a growth factor) that triggers cell division. Such molecule will directly or indirectly affect the cell that produced it as well as cells in the vicinity. This increase in the probability of cell division is symbolised in the model as a benefit.

For the dynamics we used the Moran Process^14^ which consists of the following steps: 1) at each time step, one cell is selected to reproduce according to its fitness, 2) a second cell is selected to die at random (see Fig. 2B). We studied 1 − and 2 − dimensional models using mathematical analysis and numerical simulations.

Two types of competition are considered: In global competition after a cell is chosen for growth, any cell in the compartment can be chosen for death. This scenario represents the case where physical and resource competition within the compartment does not take distance into account. In local competition, a cell within a local radius *L* of the reproducing cell is chosen for death.

We want to understand the trade-off relationships between the benefit of receiving the signal (*b*), the cost to produce it (*c*), the radius of the area affected by the signal (*R*), and the radius of the area where cell replacement takes place (*L*). To understand the role that these parameters have in the evolutionary dynamics of cancer, we calculate the fixation probability of the signalling cells for different scenarios. We investigated two variants of the model. In the simpler one, each signalling cell receives a benefit *b* and pays a cost *c* for producing the signal. This benefit is independent of the number of neighbouring signalling cells. Similarly, any non-signalling cell within the area of action of a signalling one receive a benefit *b*, independent of the number of other neighbouring signalling cells. The payoffs are, therefore “non-cumulative”. This scenario represents a case in which receptors for the signal molecule in the cell membrane are immediately saturated, or trigger a full response. In a more complex variant of the model, the payoff of both, signalling cells and non-signalling cells depends on the number of nearby signallers so that payoffs are “cumulative” (Fig. 2C).

### The stochastic process

At each time step, the number of signalling cells, *N*_*S*_ = *i*, can either increase by one, stay the same, or decrease by one. The transition probabilities from *i* to *i* + 1 and *i* − 1 are given by:

1 $${P}_{i,i+1}={B}_{S}{D}_{C}$$

2 $${P}_{i,i-1}={B}_{C}{D}_{S}$$

3 $${P}_{i,i}=1-{P}_{i,i+1}-{P}_{i,i-1}$$

Where *B*_*S*_ and *B*_*C*_ are chances of the signalling and non-signalling cells to be chosen for replication. *D*_*S*_ and *D*_*C*_ represent the probability to be chosen to die for the signalling and non-signalling cells respectively.

### The fixation probability

The process has two absorbing states *i* = 0 or *i* = *N*. Notice that for our purpose *N* does not have to be the size of the compartment. It could be that all cells in the compartment are benign, and we are interested in the chance that an emerging cancer will reach some critical size *N*. We are interested in the probability of reaching *N*_*S*_ = *N* when we start from the state *i*. To calculate the probability that *i* mutant cells ultimately take over the population, we observe that this fixation probability *a*_*i*_ follows the equation:

4 $${a}_{i}={P}_{i,i+1}\ {a}_{i+1}\ +\ {P}_{i,i}\ {a}_{i}\ -\ {P}_{i,i-1}\ {a}_{i-1}$$

with the boundary conditions *a*_0_ = 0 and *a*_*N*_ = 1.

Iterating this equation leads to the solution (^15^, p. 362^16^;):

5 $${a}_{i}=\frac{1+\mathop{\sum }\limits_{j=1}^{i-1}\mathop{\prod }\limits_{k=1}^{j}{P}_{k,k-1}/{P}_{k,k+1}}{1+\mathop{\sum }\limits_{j=1}^{N-1}\mathop{\prod }\limits_{k=1}^{j}{P}_{k,k-1}/{P}_{k,k+1}}$$

Here *P*_*k*,*k*+1_ and *P*_*k*,*k*−1_ are defined in Eqs. 1 and 2 respectively for the stochastic process.

The fixation probability, *ϕ*_*S*_( = *a*_1_) of a single signalling cell is thus

6 $${\phi }_{S}={a}_{1}=\frac{1}{1+\mathop{\sum }\limits_{j=1}^{N-1}\mathop{\prod }\limits_{k=1}^{j}\frac{{P}_{k,k-1}}{{P}_{k,k+1}}}$$

When ${P}_{k,k-1}/{P}_{k,k+1}$ is a constant, e.g. (1+*s*)^−1^, this gives the well known equation:

7 $${\phi }_{S}={a}_{1}=\frac{1}{1+\mathop{\sum }\limits_{j=1}^{N-1}{(1+s)}^{-j}}=\frac{1-{(1+s)}^{-1}}{1-{(1+s)}^{-N}},$$

which enables us to calculate the chance of the signalling cells to reach size *N*.

## Results

In the following, we present the results of mathematical analyses and numerical simulations. A full derivation of these results is presented in the Supplemental material. For the non-cumulative case, we present a full analysis of the results for a simplified model. We then present numerical simulations for comparing the non-cumulative and cumulative case. For the analysis, we studied the dynamics of the system in terms of the fitness of the signalling cells, written as (1 + *s*). Our model contains cells of only two genotypes – the signalling cells, and the wild-type cells. Some of the wild type cells will receive a benefit from diffusing signals. All the signalling cells stay in one cluster, and therefore the state of the system is fully specified by the size of the cluster, *N*_*S*_ (the number of signalling cells). We also compared our results to a cumulative system, where the benefit from different signalling cells in the vicinity adds up (Fig. 2C.II). The results do not differ significantly. In a cumulative system, the fitness of the single mutant is smaller than that of two mutants, three and so on, until the size is large enough from which point the fitness difference between mutants and non-signallers is again constant.

### Global competition, non-cumulative

In the 1D non-cumulative model with global competition, the fitness benefit of the signalling cells can be written as

8 $$1+s=1+\frac{b(1-\alpha )-c}{1+b\alpha }$$

With *α* = 2*R*∕(*N* − *N*_*s*_), the fraction of non-signalling cells receiving a benefit. The condition for *s* to be negative is

9 $$1-\alpha  < \frac{c}{b}$$

The fraction of cells that do not receive the benefit has to be smaller than *c*∕*b*. When the cost is larger than the benefit, signalling is selected against. When the cost is a fraction of the benefit, a substantial fraction of the compartment has to receive the benefit to select against signalling.

### Local competition, non-cumulative, *L*≤*R*

When *L*≤*R* all cells in the competition range receive a benefit. In the analysis, we used a simplified dynamics, where instead of competing with cells within radius *L*, cells within radius *L* of the border between signalling and non-signalling cells compete with each other (which means that all signalling cells compete when *N*_*s*_≤*L*). These allow us to get simple expressions from our analysis. In this case, our simplified dynamics is equivalent to a fitness equal to:

10 $$1+s=1+\frac{-c}{1+b}$$

The signalling cells are selected against for any *c* > 0.

### Local competition, non-cumulative, *L* > *R*

In this case, some cells in the competitive radius do not receive the benefit. Again the 1D model with the simplified dynamics is equivalent to a constant fitness difference, here with

11 $$1+s=1+\frac{(b-c)-\frac{R}{L}b}{1+\frac{R}{L}b}$$

This means that the signalling cells will be selected against when $\frac{c}{b} > \frac{L-R}{L}$. This inequality is similar to the one we encountered for global competition: the fraction of cells not receiving the benefit within the competitive radius has to be smaller than *c*∕*b*. The difference is that in this case, a large fraction of cells in the competitive radius have to receive a benefit, while in the case of global competition, a large fraction of cells in the whole compartment.

### Numerical analysis and simulations

We performed exact stochastic computer simulations for different parameter regimes. We paid particular attention to the role of the radius of action of the signalling molecule and its associated cost. For the particular case where the radius is zero (*R* = 0), the cost is zero (*c* = 0), replacement is global (*L* = *N*), and the benefit is equal to the unity (*b* = 1), the model showed the behavior of a neutral mutation, where fixation probability is 1∕*N*.

**Figure 3:**
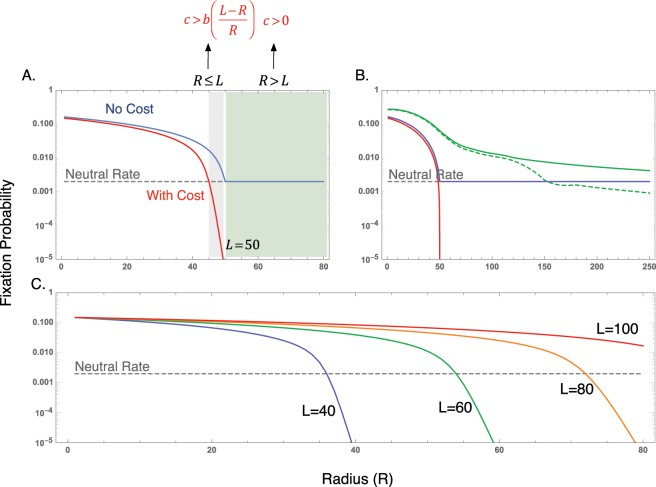
Fixation probability of the signalling cells as a function of the radius (*R*) for the one-dimensional non-cumulative model (**A**–**C**), and cumulative model (**B**). **A** The blue line indicates no cost (*c* = 0), whereas the red line indicates a positive cost (*c* = 0.2). (**B**) Cumulative model is shown in green lines. The presence of cost (*c* = 0.2) is shown with the dashed line, while absence of cost is given by the continuous line. In all cases the benefit was (*b* = 0.8). For **A**,**B**. *L* = 50. The fixation probability can be below the neutral expectation (given by the horizontal dashed line) only at large values of *R* and when the signal is costly (*c* ≠ 0) (**C**). Low values of *L* decrease the fixation probability, protecting against cancer. In all cases the system size was *N*_*T*_ = 500.

We studied the evolutionary dynamics of a mutant cell and its progeny and neglected new mutations. This assumption holds if the time to fixation of such a mutant is smaller than the time for new mutants to arise. The process has two absorbing states: either all cells are wild type, or all cells are mutated signalling cells. We performed stochastic simulations and calculated the average of 10^4^ simulations in each case and compared with the numerical solution of the equations. The results of these two were essentially the same. The fixation probability describing the evolutionary dynamics of the signalling cells is shown in Fig. 3. We observed that an increase in the radius *R* decrease the fixation probability of the mutants. Fig. 3A,B. show that some cost is required to reduce the fixation probability below the neutral expectations.

## Discussion

In the present study, we explored the conditions under which a mutant cell, expressing a molecule that enhances cell growth, could invade a population of cells. We have shown that if the molecule is costly to produce, and the benefit is given not just to the signalling cell, but to sufficient cells in the surroundings, the invasion of the mutant can be prevented. We furthermore demonstrated that when competition between cells is local, this effect is enhanced so that even if the benefit is given only to a small number of cells around the signaller, invasion of the mutant is prevented. The condition to prevent the invasion of a mutant is fulfilled, roughly, when the ratio of the cost of producing the signal to its benefit is larger than the fraction of cells not receiving the benefit among local competitors.

In our model, we compared the chance of extinction of cancer to the chance of fixation, or more precisely the probability to reach a certain critical size. Previous models studying the evolutionary dynamics of mutants producing a growth factor concluded that the population will reach a heterogeneous state in which both mutants and wild type exist^9,11^. These models assumed a diminishing return from the signals, and thus an equilibrium where both mutant and wild type exist can be stable. Our model only studies the chance for invasion. It is to be expected that as cancer grows, other factors, ignored in our simple dynamics, will change the behaviour of the tumour. The mechanisms for the prevention of the growth of the tumour would still hold.

We claim that multicellular organisms where this mechanism is present will exhibit a reduced prevalence of cancer. Is there evidence for cost and long-range effect among signalling mechanisms between cells? In the following, we give a short outline of biological evidence, and in the Supplemental material a more extended outline. A summary is provided in Table 1.

**Table 1:** Examples of direct and indirect mechanisms, factors affecting the radius and the cost.

Type of signalling	—	Sender	Effector	Radius		Cost
				Extracellular changes	Intracellular changes	
Direct	I	Wnt, Hh		Wnt^[*]^ : lipoprotein particles^28^. Heparin^30^, Heparan sulfate (HSPGs)^31–33^, SFRPs and WIFs^34–40^. Hh[*] : Dispatched^21^.	Wnt ^[1, 2]^: APC gene^41^. Hh^1^ :^42^.	Production Cost
	II	Prostaglandins		Prostaglandins^[*]^ : Kruppel-like factor (KLF)^43^.	Prostaglandins^[2]^^43^.	Production Cost. ATP-dependent exporter^44^.
**Indirect**	I	VEGF, FGF	*O*_2_, nutrients, EGE, TGF-*β*	VEGF^[*]^: Physical forces^22^. FGF[*] : Heparan sulfate^23^.	VEGF ^[1]^: EGF receptors^27,45^. TGF-β^2^ : ras, myc and fos genes. FGF^1^ :^23^.	Production Cost
	II	PDGF, TGF-*β*	IGF, HGF	TGF-*β*^[*]^: Activin^24^. HGF^[*]^: heparin and heparin sulphate^46,47^.	PDGF^[2]^ : ras, myc and fos genes^48–50^.	Production Cost

^*^Post-translational modifications and association with extracellular vehicles.

^1^Modifications in receptor number and/or affinity or ^2^modifications resulting in processes that amplify the mitogenic signal after its interaction with the receptor.

### Cost

Examples of signalling molecules triggering cell division include growth factors, cytokines, and prostaglandins. There is evidence that the production of a growth factor is costly for the cells. Archetti *et al*. directly measured in vitro the cost of producing IGF-II (a growth factor) and observed a 25% reduction in growth under some conditions^11^. The cost in our model can also represent the cost of exporting the signalling molecule. For instance, prostaglandins are exported from the cells via active transport by the prostaglandin transporter (PGT, SLCO2A1)^17^.

### Long range

It is interesting to note that growth factors are in general released out of the cell that produced them, and are then free to diffuse to other cells. Growth factors are known to exhibit both autocrine (self-stimulation) and paracrine signalling. Paracrine signalling is traditionally assumed to operate on a short-range, though there are examples of long-range diffusion. For example, Wnts, such as Wingless in Drosophila, function as concentration-dependent long-range morphogenetic signals that can act on distant neighbours^18–20^. Long-range diffusion has been observed in other growth factor families such as Hh^21^, VEGF^22^, FGF^23^ and TGF-beta^24^.

In cancer, indirect signalling for growth factors is observed where cells emit PDGFs which cause fibroblasts to release growth factors. Such mechanisms, a release of a molecule to signal for a release of a growth factor, increase the radius of effect of the original signal. If these mechanisms also exist in normal tissue, they would provide the protection outlined in our paper. Indeed, Hock *et al*.^25^ report that the release of PDGF causes an increased growth not just in the osteoblasts releasing the signal, but a general stimulation of cell growth in the bone. It is also known that PDGF signalling contributes to wound healing through stimulation of fibroblasts^26^.

Many oncogenic mutations are known to influence the radius of effect of growth factors. One example is modifying the ratio of autocrine to paracrine signalling. Thus, an increase in the number or affinity of the receptors of the cell that generated the signal or an amplification of the mitogenic signal after its interaction with the receptor^27^. A second example of effects on the radius involves changes to the concentration of extracellular modifiers of growth factors. For instance, once Wnt proteins are secreted into the extracellular matrix, a number of binding partners can modulate their activity. This means that the concentration of these binding partners will affect the range of action of the Wnt protein members^28^.

Our model suggests that in multicellular organisms signal cost and range can affect the chance of the emergence of cancer, and thus an evolutionary control of these can lower the prevalence of cancer. Many mechanisms seem to have evolved to prevent cancer – from immune system control, cell death, limits on cell proliferation, to tissue architecture. Two important questions to ask is first – why do cancer cells need to heed to the cost imposed by the organism, and second why would not the system evolve then, to fully eliminate cancer?

Cancer is an interesting parasite. In animals that go through a single-cell bottleneck to the next generation, every cancer evolves its malignant properties from scratch. Whereas the lineage evolved over millions of years to battle cancer, the cancer evolves all its properties as a parasite over the organism’s lifetime. There is thus a large asymmetry in evolutionary timescale between parasite and host. This scenario is different from the case of a regular parasite, where both evolve over many generations to battle each other. On the other hand, a cancer cell also has an advantage over a regular parasite: a cancer cell has all the genetic tools available to normal cells at its possession, whereas the regular parasite needs to evolve many of these from scratch. In our case, it means that cancer cells can produce any signal that normal cells can produce. It just needs to evolve to abuse them. However, to use those signals, it needs to use the exact genetic architecture available to the cells. Therefore, if cells pay a cost for the production of the signal, so will a cancer cell, and if the signal is a diffusible molecule, so will the cancer cell’s signal be. A cancer cell could evolve a new molecule, or alterations to the expression of several genes, to mimic the effect of the growth factor, to not require a cost, not diffuse, or maybe only be released intra-cellularly. Here the cancer would lose its benefit as a cancer, because these mechanisms are not available to regular cells, so would have to be evolved from scratch. The lineage can evolve to protect itself against these possibilities by making them cryptic, require to have several mutations, a multistage solution (Fig. 1).

Why cannot the abuse of signals be entirely prevented? When signals require cost and long range diffusion, the organism itself pays a cost. This cost could be substantial, since it is paid at every cell division, or every time the signal is used. Long range diffusion also incurs a cost, by reducing the precision of growth or response to signals. For example during development, angiogenesis, and wound healing. Thus, the prevalence of cancer will be balanced by the cost of its prevention to the organism.

Our model makes several predictions concerning signalling in multicellular organisms. We claim that signals that can be of benefit to a cancer cell will incur a cost, and will tend to diffuse or affect several cells around the signaller. Such mechanisms will be more effective in tissues with local competition. Thus, one would not expect these mechanisms to be effective in the blood. Tissues with less competition will tend to have more signalling or growth factor related cancer modifications, and tissues with local competition will evolve by overcoming the signal.

It has been suggested that costly signalling can stabilise a signalling system against abuse by cheaters^29^. Abuse is prevented if the signal is more costly to the cheaters than the benefit they would receive. Costly signalling within the multicellular organism has been suggested for neurotransmitters^12^ and for cellular competition and signals of quality within the body^13^. Is the cost of signals such as growth factors a “costly signal”? To answer this question, we need to ask what information the signal provides. An angiogenesis signal from a cell operates by requesting oxygen, inducing blood vessels’ growth in the direction where the molecular signal was generated. It is thus a signal from one part of the body to another, or the body to itself about the condition of some “regular cells" in the body. Even an intracellular or autocrine signal can be interpreted similarly. When the signal is sent by cancer cells, the response is like it is to a regular cell. Had the cancer cell provided full information to the body, stating that it is a cancer cell and that it needs a larger supply of oxygen, the multicellular body would have evolved to not respond to the signal, even when the signal is within the body. Thus, the cancer cell is cheating when it is imitating a signal. In our model, we showed how the cost and structure of the signal could cause the sending of the signal to not be beneficial for the cancer cell. In that sense, the signal can be interpreted as a costly signal. The cost to the cancer is higher than the benefit, whereas the cost to the organism is not higher than the benefit. It is an interesting case where the sender, the multicellular organism, and the cheater, the mutant cell, are not part of the same population. Costly signals between different species are not uncommon. What is special in the system is that the sender has not just control of the cost of the signal, but also of the competition regime, setting the signal up in a way that the cell competes with other cells that receive the benefit.

We have shown that multicellular organisms are not free to evolve the most efficient signalling system for development and homeostasis. Instead, they are limited by battling abuse by parasites and cancers. Signals in such organisms could evolve to incur a substantial cost.

## Supplementary information

Supplementary  Information

Supplementary Information - 2D Simulation

Supplementary Information - 3D Simulation

## Data Availability

The source code used in this study is available in the Supplementary Information.
