# Supplementary material for: Signalling architectures can prevent cancer evolution
Source: Sci Rep. 2020 Jan 27;10:674. doi: 10.1038/s41598-020-57494-w (PMC6971087; doi:10.1038/s41598-020-57494-w)
Supplement: Supplementary file 1 — Supplementary Information [file 41598_2020_57494_MOESM1_ESM.pdf]

Supplemental Material: Signalling architectures can prevent cancer evolution

Leonardo Oña, & Michael Lachmann

Contents

1 Introduction 3

2 One-Dimensional Non-Cumulative Model 3
2.1 Transition Probabilities 3
2.2 Local competition 3
2.2.1 Case $L \leq R$ 4
2.2.2 Case $L > R$ 6

3 Global competition 7

4 Two- Dimensional Model 7
4.1 Simulation 8

5 One-Dimensional Cumulative Model 8

6 Discussion of signals in multicellulars 10
6.1 Evidence for long range effects 10
6.2 Evidence for cost 13

1 Introduction

In all models presented here, a single mutant cell emerges, capable of generating a molecular signal. The signal affects the cell producing the signal and cells within a radius R. Cells producing the signal get a benefit b (constant in Sections 2-4, cumulative in Section 5) and pay a cost c for producing the signal, whereas cells in the vicinity receive the benefit b but pay no cost. The dynamic is described by a Moran process, where at each time step a cell is chosen to reproduce, and another cell chosen to be replaced. Cell replacement occurs within a radius L from the dividing cell. This method of reproduction is implemented so that mutants are always adjacent to each other. We will consider two versions of the model: one with global competition, and one with local competition. We start by analysing local competition, since the analysis is a bit simpler, and then turn to global competition.

2 One-Dimensional Non-Cumulative Model

2.1 Transition Probabilities

One key measurement for a stochastic process is the transition probability. Starting with N_S mutant cells what is the probability of increasing in number to $N_S + 1$ or decreasing to $N_S - 1$. We will start our analysis with one signalling cell ($N_S = 1$). We calculate the transition probabilities in this case and compare them to neutrality. We will then generalise our results for an arbitrary number of signalling cells.

2.2 Local competition

In local competition, we choose a cell to reproduce, weighted according to their fitness, and then choose a cell randomly to die within a radius L. This radius represents the range in which cells are in direct physical competition with each other, for example through physical forces or resource competition.

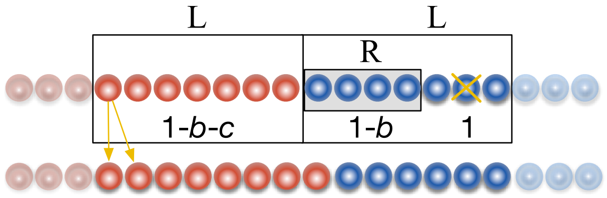

Figure S1: In the simplified model growth and death occur only within a distance L of the boundaries between the cell types. Non-signalling cells that are within distance R of the boundary receive a benefit.

Since in our model mutant cells stay in contact with one another, the boundary between the mutant signalling cells and the non-signalling cells is well defined. Therefore we analyse a simplified model where competition only occurs within a distance L of the boundary. Cells further away from this boundary might reproduce and compete, but this will not affect the number of signalling or non-signalling cells, and can, therefore, be ignored. The dynamics with competition within distance L of the boundary is not identical to the dynamics where each cell only competes with cells within distance L of itself, but the results are very similar, and the analysis is more straightforward. Within the compartment, there can be three types of cells: signalling cells receiving benefits and paying a cost for signalling, non-signalling cells receiving a benefit but not paying a cost, and cells not affected by the signal having a basal fitness equal to 1.

We need to distinguish the cases where $L \leq R$, which means that all cells competing with a signalling cell receive a benefit, from $L > R$, where in the competition region some cells receive a benefit, and some do not.

2.2.1 Case $L \leq R$

The Moran process gives us a random walk in the number of signalling cells, N_S. In the random walk, the ratio of the chance to increase N_S to the chance to decrease N_S is relevant. For simple selection with mutants that have a

fitness of $1+s$ vs. wild type with fitness 1, the ratio would be $(1+s)$.

The number of signalling cells will increase if we select a signalling cell for reproduction, and a non-signalling cell for death, and vice versa for a decrease. If B_S is the probability to select a signalling cell for reproduction, D_C is the probability of a non-signalling cell to die, B_C is the probability of selecting a non-signalling cell for reproduction, and D_S is the probability of selecting a signalling cell to die, b is the benefit of receiving the signal, and c the cost of producing it, then the chance to increase can be written as (first assuming $N_S > L$):

$$P_{N_S,N_S+1} = \overbrace{\frac{L(1+b-c)}{L(1+b-c)+L(1+b)}}^{B_S} \cdot \overbrace{\frac{L}{2L}}^{D_C} \tag{1}$$

The chance to decrease is:

$$P_{N_S,N_S-1} = \overbrace{\frac{L(1+b)}{L(1+b-c)+L(1+b)}}^{B_S} \cdot \overbrace{\frac{L}{2L}}^{D_C} \tag{2}$$

The ratio between these two is:

$$\frac{P_{N_S,N_S+1}}{P_{N_S,N_S-1}} = \frac{1+b-c}{1+b} = 1 - \frac{c}{1+b} \tag{3}$$

We see that the ratio is < 1 when $c > 0$. We see that the dynamics of the system is like a system with a genotype with a fitness detriment of $s = -c/(1+b)$.

When $N_S < L$, we get:

$$P_{N_S,N_S+1} = \overbrace{\frac{N_S(1+b-c)}{N_S(1+b-c)+2L(1+b)}}^{B_S} \cdot \overbrace{\frac{2L}{N_S+2L}}^{D_C}. \tag{4}$$

The chance to decrease is

$$P_{N_S,N_S-1} = \overbrace{\frac{2L(1+b)}{N_S(1+b-c)+2L(1+b)}}^{B_S} \cdot \overbrace{\frac{N_S}{N_S+2L}}^{D_C} \tag{5}$$

giving again, the same ratio. The chance to fix is, therefore, the same chance as for a regular Moran process with a fitness of $s = -c/(1+b)$.

2.2.2 Case $L > R$

Now there will be three types of cells. We again start with $N_S > L$.

$$P_{N_S,N_S+1} = \overbrace{\frac{L(1+b-c)}{L(1+b-c)+R(1+b)+(L-R)\cdot 1}}^{B_S} \cdot \overbrace{\frac{L}{2L}}^{D_C} \quad (6)$$

$$P_{N_S,N_S-1} = \overbrace{\frac{R(1+b)+(L-R)\cdot 1}{L(1+b-c)+R(1+b)+(L-R)\cdot 1}}^{B_S} \cdot \overbrace{\frac{L}{2L}}^{D_C} \quad (7)$$

And their ratio is

$$\frac{P_{N_S,N_S+1}}{P_{N_S,N_S-1}} = \frac{L(1+b-c)}{R(1+b)+(L-R)} = 1 + \frac{(b-c)-\frac{R}{L}b}{1+\frac{R}{L}b} \quad (8)$$

Again we have a dynamics that is equivalent to constant selection, with a selection pressure that also depends on R. Selection will be negative when $\frac{b}{c} < \frac{L}{L-R}$, which can give us a lower bound both on R or c.

When $N_S < L$ we get

$$P_{N_S,N_S+1} = \overbrace{\frac{N_S(1+b-c)}{N_S(1+b-c)+2R(1+b)+2(L-R)\cdot 1}}^{B_S} \cdot \overbrace{\frac{2L}{2L+N_S}}^{D_C} \quad (9)$$

$$P_{N_S,N_S-1} = \overbrace{\frac{2R(1+b)+2(L-R)\cdot 1}{N_S(1+b-c)+2R(1+b)+2(L-R)\cdot 1}}^{B_S} \cdot \overbrace{\frac{N_S}{2L+N_S}}^{D_C} \quad (10)$$

Giving again, the same ratio. Again the system will behave exactly like a system with a mutant with a constant s.

3 Global competition

Now all N cells in the compartment compete. Of the non-signalling cells, R receive a benefit, and $N - 2R - N_S$ do not.

$$\begin{aligned}
P_{N_S,N_S+1} &= \overbrace{\frac{N_S(1+b-c)}{N_S(1+b-c)+2R(1+b)+(N-2R-N_S)\cdot 1}}^{B_S} \cdot \overbrace{\frac{N-N_S}{N}}^{D_C} \\
P_{N_S,N_S-1} &= \overbrace{\frac{2R(1+b)+(N-2R-N_S)\cdot 1}{N_S(1+b-c)+2R(1+b)+(N-2R-N_S)\cdot 1}}^{B_S} \cdot \overbrace{\frac{N_S}{N}}^{D_C} \qquad (11)
\end{aligned}$$

Giving a ratio of:

$$\frac{P_{N_S,N_S+1}}{P_{N_S,N_S-1}} = \frac{(N-N_S)(1+b-c)}{2R(1+b)+(N-2R-N_S)} \qquad (12)$$

We see that with global competition the selection pressure is dependent on N_S, so the system is not equivalent to a system with constant selection pressure. We can, however, look at the direction of selection. If we rewrite the above expression as $1+s$, we get

$$s = \frac{b(1-\alpha)-c}{1+b\alpha} \qquad (13)$$

with $\alpha = 2R/(N-N_S)$. If the range of benefit given, i.e., R, is very small, the cost has to be larger than the benefit to have negative selection. On the other hand, when α is close to 1, a positive cost results in negative selection. We see that in non-local competition the benefit has to be given to a large fraction of cells to prevent the invasion of the mutant.

4 Two- Dimensional Model

In two dimensions a cell will compete with cells within a radius L. An assumption equivalent to the analysis done above with competition within a range L of the boundary is competition within distance L perpendicular to the boundary, and L along the boundary (see Figure S2). The analysis is then identical to the one-dimensional case.

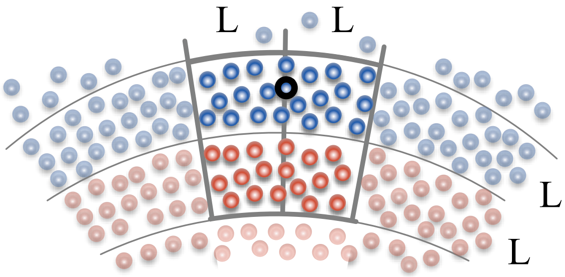

Figure S2: Simplified 2D model. Only cells within a distance L of the boundary are taken into account for reproduction. Other could reproduce, but that will not affect the frequency of the cell types. When the indicated cell reproduces it can replace any cell in the competition region – a distance L from the boundary, and L along the boundary.

4.1 Simulation

We also simulated 2D models. signalling cells have a fitness of $(1 + b - c)$. Any non-signalling cell within a distance R of a signalling cell has a fitness of $(1+b)$, and all the remaining non-signalling cells a fitness equal to 1. Cells are chosen to reproduce according to fitness. Then a random cell within a radius L is chosen for death. If that cell is of the opposite type of the reproducing cell, then a cell of the same type on the boundary between the two types is removed and replaced by a cell of the reproducing type.

5 One-Dimensional Cumulative Model

In the non-cumulative model, each cell receives the same amount of benefit independently of the number of signalling cells present. In the cumulative model, the amount of benefit that each cell receives does depend on the number of signalling cells. As the number of signalling cells grows, such benefit will accumulate, and therefore the average fitness of each cell type will be frequency dependent. In order to calculate the transition probabilities,

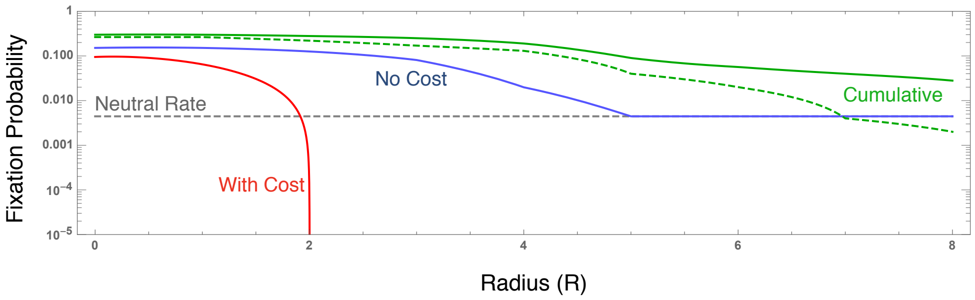

Figure S3: Fixation probability of the signalling cells as a function of the radius (R) for the two-dimensional model. Non-cumulative model is given by the blue line for no cost ($c = 0$), whereas the red line indicate a positive cost ($c = 0.09$). Cumulative model is shown in green lines. The presence of cost ($c = 0.09$) is shown with the dashed line, while absence of cost is given by the continuous line. In all cases the benefit was ($b = 0.2$), and $L = 5$. The size of the system is 15x15 cells.

we need an analytical expression for the total payoff of each cell type.

We find two regimes according to a relationship between the number of N_S cells and radius R. Such regimes are determined by the formation of a "cluster" of N_S cells, defined by an amount of N_S cells, such as the benefit from a cell in one extreme of the cluster (in one extreme of the one dimensional arrangement of cells) cannot reach a N_C cell in the other extreme. Then, the cluster will be present when $N_S \geq (R+1)$.

Before the cluster is formed ($N_S < (R+1)$), each N_S cell will receive N_S units of benefit, therefore the total amount of benefit that N_S cells receive is given by N_S^2. On the other hand, N_C cells receive a benefit equal to R from the N_S cell from the extreme, $(R-1)$, from the N_S cell from the next one, and so on, and the same pattern in the other border. In this way, the total amount of benefit for the N_C cells will be $\sum_{i=1}^{R} i - \sum_{i=1}^{R-N_S} i = N_S\,(1 + 2R - N_S)$. After the cluster is formed ($N_S \geq (R+1)$), the first N_C cell closer to the N_S cells will receive R unities of benefit, the second N_C cell will receive $(R-1)$

unities of benefit and so on. As this pattern is present at both sides of the N_S cluster, we obtain that the total payoff for N_C cells is $\sum_{i=1}^{R} i = R(R+1)$. In a similar way one can show that, after the cluster is formed, N_S cell will receive a total payoff equal to $R(2N_S - R - 1) + N_S$.

Summarising, for the signalling cells the amount of benefit they will receive:

- If $N_S \leq (R+1)$ is N_S^2
- If $N_S > (R+1)$ is $R(2N_S - R - 1) + N_S$

For the non-signalling cells, the benefit they will receive:

- If $N_S \leq (R+1)$ is $N_S(1 + 2R - N_S)$
- If $N_S > (R+1)$ is $R(R+1)$

The interplay between the number of signalling cells N_S, the radius R and the level of locality of the competition L generates scenarios that are not easy to tackle analytically. We performed numerical simulations to study the evolutionary output of the interaction between these variables and parameters.

6 Discussion of signals in multicellulars

6.1 Evidence for long range effects

Although it has been traditionally assumed that growth factors signal through paracrine signalling, operating in rather short range diffusion, evidence shows that growth factors can exhibit long-range diffusion. Wnts, such as Wingless in *Drosophila*, function as concentration-dependent long-range morphogenetic signals that can act on distant neighbours [6, 38, 44]. It has been proposed that Wnt is vesicle-based transported outside of cells in Drosophila wing imaginal discs. The vesicles, termed argosomes, might carry Wg protein (Wg in *Drosophila* is the homologue of Wnt1 in mammals) as cargo [17].

Wnts may also be transported by cytonemes, which are long, thin filopodial processes that might carry Wnts and other growth factors away from signalling cells [31]. Moreover, as mentioned in the main text, long-range diffusion has been observed in other growth factors families such as Hh [7], VEGF [33], FGF [16] and TGF-beta [21].

The main result of the present study is that long-range diffusion (large radius R) can decrease the probability of fixation of a mutant cell sending a mitogenic signal, which influences the region around where the signal was generated. In this context, any biological process affecting the radius will directly have an impact on the fitness of the mutant cell. Different biological processes can affect the radius. These include intracellular changes in the mutant cell, favouring the autocrine signalling, or extracellular changes increasing the diffusion of the mitogenic signal.

Intracellular changes favouring autocrine signalling will increase the risk of cancer in cases where the mitogenic signal operates directly, through an autocrine and paracrine mechanism (direct mechanism). Autocrine signalling can be favoured by increasing the number or affinity of the receptors of the cell that generated the signal or by amplifying the mitogenic signal after its interaction with the receptor. In the last case, the signalling pathway may be modified by oncogene expression at the receptor or post-receptor levels [37]. As an example, an increase at very high number in EGF receptors of the TGF-α occur in squamous carcinoma and A431 cell lines both from human head and neck cancers [37, 12]. Another type of tumour cell in which receptor abnormality can generate cancer is the leukemic adult T cell, where unregulated expression of the receptor for interleukin-2 (IL-2, T-cell growth factor) contribute to leukemogenesis [37, 8]. The oncogenes ras, myc and fos are examples of genes that confer growth factor autonomy via indirect mechanisms, such as the post-receptor signal transduction pathways that generate a mitogenic response in a cell after growth factor stimulation: that is, the events whereby changes in the membrane receptor are translated into activation of specific genes in the nucleus [37]. Several lines of evidence suggest that

the genes ras, myc and fos affect the signalling effects of TGF-α [14, 20, 45] and PDGF [18, 5, 43] growth factors. Another example of a process that activates a post-receptor signalling mechanism and leads eventually to a mitogenic response present in the Wnt is the one involving the APC gene. The canonical Wnt pathway (or Wnt/β-catenin pathway) is the Wnt pathway that causes an accumulation of a protein called β-catenin in the cytoplasm and its eventual translocation into the nucleus to act as a transcriptional coactivator of transcription factors and triggers a mitogenic response. The APC gene is known for its role in colon cancer [30, 29]: the most common mutation in colon cancer causes the inactivation of APC. The APC gene is a tumour suppressor gene (which mean that it is normally expressed, and prevent the uncontrolled growth of cells that may result in cancerous tumours). The APC protein controls the activity of β-catenin. The APC protein builds a complex with other proteins. This complex is then able to bind β-catenins in the cytoplasm, which results in β-catenin ubiquitination and degradation by cellular proteosomes. This prevents it from translocating into the nucleus, where it acts as a transcription factor for proliferation genes [24].

Extracellular changes increasing the diffusion of the mitogenic signal can directly affect the radius. Different processes modulate the radius of action of Wnt proteins and their exportation to the extracellular matrix. This includes specific post-translational modifications, the controlled sorting of Wnt to specialised subcellular compartments and its association with extracellular vehicles [11]. For instance, it has been shown that the production, secretion, and release of the Wnt signal depends on Wntless (wls) [3] or evenness interrupted (evi) [4]: a gene that encodes a multipass transmembrane protein that is conserved in metazoans from *C. elegans* to humans. Once Wnt proteins are secreted, many binding partners can modulate their activity. Wnt paracrine signalling also depends on its association with lipoprotein particles [11], Heparin [22] and Heparan sulfate (HSPGs) [27]. Emerging evidence suggests a role for HSPGs in the transport or stabilization of Wnt. In *Drosophila*, absence of a particular HSPG [27, 39], and mutations in genes

encoding enzymes that modify HSPG [1, 27] generate phenotypes similar to wingless mutants. Some authors have postulated that HSPGs function as coreceptors on target cells [27], while others suggested that HSPGs may stabilise Wnt proteins or aid in its presentation or movement between cells [28]. Other regulators of Wnt radius include proteins of the SFRP and WIF families. SFRPs and WIFs are thought to function as extracellular Wnt inhibitors [2, 13, 19, 26, 34, 41, 42].

6.2 Evidence for cost

Finally, a crucial parameter in our model is the cost (c). The production of any molecule represents a physiological cost to a cell. Also, this cost can represent the cost of exporting the molecule. For instance, prostaglandins are exported from the cells via active transport by the prostaglandin transporter (PGT, SLCO2A1) [23] and by the multi-drug resistance protein 4 (MRP4, ABCC4) [32], a member of the ATP-binding cassette transporter superfamily. Therefore, the exportation of prostaglandins is an (ATP) energy-consuming process. Although we mentioned the role of prostaglandins operating through a direct mechanism increasing cell proliferation [15, 10, 25, 36], they also operate through an indirect mechanism increasing tumour angiogenesis [36, 40, 9, 35].

References

[1] G. H. Baeg, X. Lin, N. Khare, S. Baumgartner, and N. Perrimon. Heparan sulfate proteoglycans are critical for the organization of the extracellular distribution of Wingless. *Development*, 128(1):87–94, Jan 2001.

[2] A. Bafico, A. Gazit, T. Pramila, P. W. Finch, A. Yaniv, and S. A. Aaronson. Interaction of frizzled related protein (FRP) with Wnt ligands and the frizzled receptor suggests alternative mechanisms for FRP inhibition of Wnt signaling. *J. Biol. Chem.*, 274(23):16180–16187, Jun 1999.

[3] C Bänziger, D Soldini, C Schütt, P Zipperlen, G Hausmann, and K Basler. Wntless, a conserved membrane protein dedicated to the secretion of wnt proteins from signaling cells. *Cell*, 125(3):509–522, 2006.

[4] K Bartscherer, N Pelte, D Ingelfinger, and M Boutros. Secretion of wnt ligands requires evi, a conserved transmembrane protein. *Cell*, 125(3):523–533, 2006.

[5] R. A. Blake, M. A. Broome, X. Liu, J. Wu, M. Gishizky, L. Sun, and S. A. Courtneidge. SU6656, a selective src family kinase inhibitor, used to probe growth factor signaling. *Mol. Cell. Biol.*, 20(23):9018–9027, Dec 2000.

[6] K. M. Cadigan, M. P. Fish, E. J. Rulifson, and R. Nusse. Wingless repression of Drosophila frizzled 2 expression shapes the Wingless morphogen gradient in the wing. *Cell*, 93(5):767–777, May 1998.

[7] A. Callejo, A. Bilioni, E. Mollica, N. Gorfinkiel, G. Andres, C. Ibanez, C. Torroja, L. Doglio, J. Sierra, and I. Guerrero. Dispatched mediates Hedgehog basolateral release to form the long-range morphogenetic gradient in the Drosophila wing disk epithelium. *Proc. Natl. Acad. Sci. U.S.A.*, 108(31):12591–12598, Aug 2011.

[8] J Chen, M Petrus, BR Bryant, VP Nguyen, CK Goldman, R Bamford, JC Morris, and JE Janik Jand TA Waldmann. Autocrine/paracrine cytokine stimulation of leukemic cell proliferation in smoldering and chronic adult t-cell leukemia. *Blood*, 116(26):5948–5956, 2010.

[9] V Chiarugi, L Magnelli, and O Gallo. Cox-2, inos and p53 as playmakers of tumor angiogenesis. *Int J Mol Med*, 2(6):715–719, 1998.

[10] RJ Coffey, CJ Hawkey, L Damstrup, R Graves-Deal, VC Daniel, PJ Dempsey, R Chinery, SC Kirkland, RN DuBois, TL Jetton, and JD Morrow. Epidermal growth factor receptor activation induces nuclear targeting of cyclooxygenase-2, baso lateral release of prostaglandins and

mitogenesis in polarizing colon cancer cells. *Proc Natl Acad Sci U S A.*, 94(2):657–662, 1997.

[11] D Coudreuse and HC Korswagen. The making of wnt: new insights into wnt maturation, sorting and secretion. *Development*, 134(1):3–12, 2007.

[12] G Cowley, JA Smith, BA Gusterson, FJ Hendler, and B Ozanne. The amount of egf receptor is elevated on squamous cel carcinomas. *Cancer Cells*, 1:5–10, 1984.

[13] S. Dennis, M. Aikawa, W. Szeto, P. A. d'Amore, and J. Papkoff. A secreted frizzled related protein, FrzA, selectively associates with Wnt-1 protein and regulates wnt-1 signaling. *J. Cell. Sci.*, 112 (Pt 21):3815–3820, Nov 1999.

[14] X. H. Feng, Y. Y. Liang, M. Liang, W. Zhai, and X. Lin. Direct interaction of c-Myc with Smad2 and Smad3 to inhibit TGF-beta-mediated induction of the CDK inhibitor p15(Ink4B). *Mol. Cell*, 9(1):133–143, Jan 2002.

[15] SM Fischer. Prostaglandins and cancer [reviews: Basic science]. *Front Biosci*, 2:482–500, 1997.

[16] R. Goetz and M. Mohammadi. Exploring mechanisms of FGF signalling through the lens of structural biology. *Nat. Rev. Mol. Cell Biol.*, 14(3):166–180, Mar 2013.

[17] V. Greco, M. Hannus, and S. Eaton. Argosomes: a potential vehicle for the spread of morphogens through epithelia. *Cell*, 106(5):633–645, Sep 2001.

[18] R. Hernandez-Alcoceba, L. del Peso, and J. C. Lacal. The Ras family of GTPases in cancer cell invasion. *Cell. Mol. Life Sci.*, 57(1):65–76, Jan 2000.

[19] J. C. Hsieh, L. Kodjabachian, M. L. Rebbert, A. Rattner, P. M. Smallwood, C. H. Samos, R. Nusse, I. B. Dawid, and J. Nathans. A new secreted protein that binds to Wnt proteins and inhibits their activities. *Nature*, 398(6726):431–436, Apr 1999.

[20] E. Janda, K. Lehmann, I. Killisch, M. Jechlinger, M. Herzig, J. Downward, H. Beug, and S. Grunert. Ras and TGF[beta] cooperatively regulate epithelial cell plasticity and metastasis: dissection of Ras signaling pathways. *J. Cell Biol.*, 156(2):299–313, Jan 2002.

[21] C. M. Jones, N. Armes, and J. C. Smith. Signalling by TGF-beta family members: short-range effects of Xnr-2 and BMP-4 contrast with the long-range effects of activin. *Curr. Biol.*, 6(11):1468–1475, Nov 1996.

[22] SF Jue, RS Bradley, JA Rudnicki, HE Varmus, and AM Brown. The mouse wnt-1 gene can act via a paracrine mechanism in transformation of mammary epithelial cells. *Mol Cell Biol*, 12(1):321–328, 1992.

[23] N Kanai, R Lu, JA Satriano, Y Bao, AW Wolkoff, and VL Schuster. Identification and characterization of a prostaglandin transporter. *Science*, 268(5212):866–869, 1995.

[24] A Klaus and W Birchmeier. Wnt signalling and its impact on development and cancer. *Nat Rev Cancer.*, 8(5):387–398, 2008.

[25] GN Levy. Prostaglandin-h synthase, non steroidal anti-inflammatory drugs and colon cancer. *The FASEB Journa*, 11:234–247, 1997.

[26] L. Leyns, T. Bouwmeester, S. H. Kim, S. Piccolo, and E. M. De Robertis. Frzb-1 is a secreted antagonist of Wnt signaling expressed in the Spemann organizer. *Cell*, 88(6):747–756, Mar 1997.

[27] X. Lin and N. Perrimon. Dally cooperates with Drosophila Frizzled 2 to transduce Wingless signalling. *Nature*, 400(6741):281–284, Jul 1999.

[28] L. Lum, S. Yao, B. Mozer, A. Rovescalli, D. Von Kessler, M. Nirenberg, and P. A. Beachy. Identification of Hedgehog pathway components by RNAi in Drosophila cultured cells. *Science*, 299(5615):2039–2045, Mar 2003.

[29] SD Markowitz and MM Bertagnolli. Molecular basis of colorectal cancer. *N. Engl. J. Med.*, 361(25):2449–2460, 2009.

[30] I Nishisho, Y Nakamura, Y Miyoshi, Y Miki, H Ando, A Horii, K Koyama, J Utsunomiya, S Baba, and P Hedge. Mutations of chromosome 5q21 genes in fap and colorectal cancer patients. *Science*, 253(5020):665–669, 1991.

[31] F. A. Ramirez-Weber and T. B. Kornberg. Cytonemes: cellular processes that project to the principal signaling center in Drosophila imaginal discs. *Cell*, 97(5):599–607, May 1999.

[32] G Reid, P Wielinga, N Zelcer, I van der Heijden, A Kuil, M de Haas, J Wijnholds, and P Borst. The human multidrug resistance protein mrp4 functions as a prostaglandin efflux transporter and is inhibited by nonsteroidal antiinflammatory drugs. *Proc Natl Acad Sci U S A*, 100(3):9244–9249, 2003.

[33] N. C. Rivron, E. J. Vrij, J. Rouwkema, S. Le Gac, A. van den Berg, R. K. Truckenmuller, and C. A. van Blitterswijk. Tissue deformation spatially modulates VEGF signaling and angiogenesis. *Proc. Natl. Acad. Sci. U.S.A.*, 109(18):6886–6891, May 2012.

[34] A. N. Salic, K. L. Kroll, L. M. Evans, and M. W. Kirschner. Sizzled: a secreted Xwnt8 antagonist expressed in the ventral marginal zone of Xenopus embryos. *Development*, 124(23):4739–4748, Dec 1997.

[35] H Sawaoka, S Tsuji, M Tsujii, ES Gunawan, Y Sasaki, S Kawano, and M Hori. Cyclooxygenase inhibitors suppress angiogenesis and reduce tumor growth in vi. *Lab Invest*, 79(12):1469–1477, 1999.

[36] E Spisni and V Tomasi. Involvement of prostanoids in angiogenesis in: Bicknell roy, lewis claire e and ferrara napoleone, editors. tumor angiogenesis. *Tumor Angiogenesis. 1st ed. NewYork: Oxford University Press*, pages 291–300, 1997.

[37] MB Sporn and AB Roberts. Autocrine growth factors and cancer. *Nature*, 313(6005):745–747, 2001.

[38] M. Strigini and S. M. Cohen. Wingless gradient formation in the Drosophila wing. *Curr. Biol.*, 10(6):293–300, Mar 2000.

[39] M. Tsuda, K. Kamimura, H. Nakato, M. Archer, W. Staatz, B. Fox, M. Humphrey, S. Olson, T. Futch, V. Kaluza, E. Siegfried, L. Stam, and S. B. Selleck. The cell-surface proteoglycan Dally regulates Wingless signalling in Drosophila. *Nature*, 400(6741):276–280, Jul 1999.

[40] K Uefuji, T Ichikura, and H Mochizuki. Cyclooxygenase-2 ex- pression is related to prostaglandin biosynthesis and angiogenesis in human gastric cancer. *Clin Cancer Res.*, 6(1):135–138, 2000.

[41] A. Uren, F. Reichsman, V. Anest, W. G. Taylor, K. Muraiso, D. P. Bottaro, S. Cumberledge, and J. S. Rubin. Secreted frizzled-related protein-1 binds directly to Wingless and is a biphasic modulator of Wnt signaling. *J. Biol. Chem.*, 275(6):4374–4382, Feb 2000.

[42] S. Wang, M. Krinks, K. Lin, F. P. Luyten, and M. Moos. Frzb, a secreted protein expressed in the Spemann organizer, binds and inhibits Wnt-8. *Cell*, 88(6):757–766, Mar 1997.

[43] H. Q. Yao, Y. Peng, Z. Z. Zhong, H. X. He, and Z. H. Li. Association of the expressions of platelet-derived growth factor receptor and c-Fos with the biological characteristics of bladder cancer. *Di Yi Jun Yi Da Xue Xue Bao*, 24(2):177–179, Feb 2004.

[44] M Zecca, K Basler, and G Struhl. Direct and long-range action of a wingless morphogen gradient. *Cell*, 87(5):833–844, 1996.

[45] Y. Zhang, X. H. Feng, and R. Derynck. Smad3 and Smad4 cooperate with c-Jun/c-Fos to mediate TGF-beta-induced transcription. *Nature*, 394(6696):909–913, Aug 1998.
