# Supplementary material for: Signalling architectures can prevent cancer evolution
Source: Sci Rep. 2020 Jan 27;10:674. doi: 10.1038/s41598-020-57494-w (PMC6971087; doi:10.1038/s41598-020-57494-w)
Supplement: Supplementary file 3 — Supplementary Information - 3D Simulation [file 41598_2020_57494_MOESM3_ESM.pdf]

1. Introduction
2. Main program
3. includes
4. Parameters
5. Functions
6. Fitness and replacement

3D simulation

1. Introduction

The program simulates a compartment with possible cancer cells by iterating steps of the Moran process. Each run starts from a single mutation till fixation. One modification to the regular Moran is that cells to replace are chosen so that the lump of mutants stays compact.j

For more information about compiling this program, see https://github.com/mic-lachmann/SignalingCancer

2. Main program

2.1. The Moran process

Let us start with an overview of the implementation of the Moran Process.

In each single run first initialise the full compartment, stored in `MatrixState` of size `iSize*jSize*kSize`, to type 0, and set one single cell (the middle one) to type 1.

2.1.1. initialise compartment and introduce single mutant

{initialise compartment and introduce single mutant 2.1.1}

```
bool MatrixState [ iSize   ][ jSize   ][ kSize  ] = {{{ 0 }}} ;
MatrixState [ iSize/2 ][ jSize/2 ][ kSize/2]   =   1   ;   // Mutant
```

Used in section 2.1.3

2.1.2. Single moran step

Then at each Moran step choose a cell to replicate, the *reproducer*, and a cell that will be replaced.

{Single moran step 2.1.2}

```
{Choose *reproducer* according to fitness, 6.1}
{Choose random cell within range L *to be replaced*, 6.2}
{Find cell of the same type as *to be replaced*, having maximum neigbours of same type as the *reproducer*, and
replace it instead, 6.3}
```

Used in section 2.1.3

Note that finding the cell to be replaced has two stops: first chose one in radius L, then chose an identical one anywhere that has a maximal number of neighbours of the same type as the reproducer.

2.1.3. main loop single run

This is done till the mutant either is extinct, or takes up the whole space. `call_count` records how long this took.

{main loop single run 2.1.3}

```
  {initialise compartment and introduce single mutant, 2.1.1}
  static unsigned int call_count = 0;
  do {
    {Single moran step, 2.1.2}
    call_count++ ;
  } while( SumArr3(MatrixState, iSize, jSize, kSize) != 0     &&
           SumArr3(MatrixState, iSize, jSize, kSize) != PopSize    );
         // Notice that here SumArr3 is called twice. Also, in a single step, only one replacement takes
         // place, and yet the 6x6x6 sum is calculated again (twice)
```

Used in section 3.1

2.1.3.1. definition of PopSize

Notice that `PopSize` is set to the size of the array

{definition of PopSize 2.1.3.1}

```
const int PopSize = iSize*jSize*kSize;
```

Used in section 4

2.2. Main program for 3D run

The program has a very simple structure, of a main loop, with inits and printing results.

2.2.1. main3.cpp

{**main3.cpp** 2.2.1}

```
//
//  main.cpp
//  Cancer3D
//
//  Created by Leonardo Ona Bubach on 04/11/2015.
//  Copyright © 2015 Leonardo Ona Bubach. All rights reserved.
//

{includes, 3}
{Parameters, 4}
{Functions, 5}
int main() {
   ofstream outputfile ("output.csv");

   {main program inits, 5.1.1}

   {main loop stats init, 3.2.2}

   {main loop, 3.1}

   outputfile.close();

   return 0;
}
```

3. includes

We will gather includes as we need them over the program. `<fstream>` is needed for `ofstream`.

{includes 3}

```
#include <fstream>
```

Added to in sections 3.2.4.0.1, 5.0.1.0.1, 6.1.1.9.4, 6.3.1.1.1, 6.3.1.2.1 and 6.3.1.4.1

Used in section 2.2.1

3.1. main loop

The main loop runs across different parameters. In the current incarnation, it runs across parameter `R`, and for each value of `R` runs `nRuns` runs. The arrays `whoWon[]` and `fixTime[]` will collect results on each single run.

{main loop 3.1}

```
for (int R=Rmin; R <= Rmax ; R=R+Rstep) {

  int whoWon[nRuns]={0};
  int fixTime[nRuns]={0};

  for (int i=0; i<nRuns; i++){
    {main loop single run, 2.1.3}
    {record who won and fixation time, 3.2.1}
  }

   {collect stats and print results, 3.2.3}
}
```

Used in section 2.2.1

3.2. Stats and printout

3.2.1. record who won and fixation time

After the run record who fixed by sampling a single cell, and record time to fixation from `call_count`.

{record who won and fixation time 3.2.1}

```
whoWon[i]  = MatrixState[1][1][1];
fixTime[i] = call_count;
```

Used in section 3.1

During the actual main loop, `whoWon` and `fixTime` recorded results. These are converted to the statistics we actually want to print out. These are the fixation probability, and average time to fixation, conditional on fixation.

3.2.2. main loop stats init

{main loop stats init 3.2.2}

```
double fixProbR[ nR ] ={ 0.0 };
double avFixTimeR[ nR ]={ 0.0 };
```

Used in section 2.2.1

We loop over all runs, and for cases in which the mutant fixed, record time to fixation in `OnlyCasesOneFixTime[]`. All other entries stay 0. `Counter1` records how often this happened.

3.2.3. collect stats and print results

{collect stats and print results 3.2.3}

```
//****The following vector save cases where 1 reach fixation*****

int OnlyCasesOneFixTime[nRuns]={0};

int Counter1=0;

for (int i=0; i<nRuns; i++) {
  if(whoWon[i]==1){ // mutant fixed
    Counter1++;
    OnlyCasesOneFixTime[i]=fixTime[i];
  }
 } // End of the For loop for Runs values
//******************************************************************
```

Added to in section 3.2.4

Used in section 3.1

Now we just need

3.2.4. collect stats and print results +=

{collect stats and print results 3.2.3} +=

```
fixProbR[   (R-Rmin) / Rstep ]= (double(Sum( whoWon,              nRuns))/nRuns);
avFixTimeR[ (R-Rmin) / Rstep ]= (double(Sum( OnlyCasesOneFixTime, nRuns))/Counter1)/PopSize;


cout << endl;
cout << endl;


cout << "Radius:"<< endl;
for (int i= Rmin; i <= Rmax; i= i+Rstep) {
  cout << i << " ";
}

cout << endl;
cout << endl;



cout << "Fix Prob dep R:"<< endl;
for (int i=0; i<=(Rmax-Rmin)/Rstep; i++) {
  cout << fixProbR[i] << " ";
 }

cout << endl;
cout << endl;

cout << "Av Fix Time dep R:"<< endl;
for (int i=0; i<=(Rmax-Rmin)/Rstep; i++) {
  cout << avFixTimeR[i] << " ";
}



cout << endl;
cout << endl;
cout << endl;



for (int i=0; i<=(Rmax-Rmin)/Rstep; i++)  {
  outputfile << i << "," << fixProbR[i] << "," << avFixTimeR[i] << endl;
}
```

Used in section 3.1

3.2.4.0.1. includes +=

Using `cout` means we need

{includes 3} +=

```
#include <iostream>
```

Added to in sections 5.0.1.0.1, 6.1.1.9.4, 6.3.1.1.1, 6.3.1.2.1 and 6.3.1.4.1

Used in section 2.2.1

4. Parameters

{Parameters 4}

```
using namespace std ;
//PARAMETERS

#define CUMULATIVE

const double b=1.0;                             // Benefit
const double c=0.01;                            // Cost
//const int R=30;                               // Radius
const int Rmin=0;                               // Min Radius in a loop
const int Rmax=3;                               // Max Radius in a loop
const int Rstep=1;                              // R step in a loop
const int nR= ((Rmax-Rmin)/Rstep)+1;            // Number of data per Radius
const int L=1;                                  // Range of local competition L

const int iSize = 6;
const int jSize = 6;
const int kSize = 6;

{definition of PopSize, 2.1.3.1}

const int nRuns=100;                            // Number of runs


int MapVM[iSize][jSize][kSize]= {{{ 0 }}} ;
```

Used in section 2.2.1

5. Functions

{Functions 5}

```
{IntegerRandom, 5.0.1}
{Sum, 5.0.2}
{SumArr3, 5.0.3}
{WithinCompartment, 5.0.4}
{MatMappingInitialization, 5.1.1.1}
{MatToVet, 5.1.1.2}
{VetToMat, 5.1.2}
{ChooseAnElement, 6.1.1}
{NeighbourWithinL, 6.2.1}
{Replacement, 6.3.1}
```

Used in section 2.2.1

5.0.1. IntegerRandom

{IntegerRandom 5.0.1}

```
//The function "IntegerRandom" returns a random integer from min (inclusive) to max (exclusive): [min,max). When
called with one argument gives [0,max)
int IntegerRandom(const int max, const int min=0)
{
  return static_cast<int>( rnd::uniform()*(max-min)+min );
}
```

Used in section 5

`rnd` is defined in `random.h`

5.0.1.0.1. includes +=

{includes 3} +=

```
#include "random.h" // for rnd::uniform()
```

Added to in sections 3.2.4.0.1, 6.1.1.9.4, 6.3.1.1.1, 6.3.1.2.1 and 6.3.1.4.1

Used in section 2.2.1

5.0.2. Sum

{Sum 5.0.2}

```
int Sum(const int *pnArray, const int nLength)
{
    int val =0;
    for ( int i=0; i<nLength; i++)
        val += pnArray[i];
    return val;
}
```

Used in section 5

5.0.3. SumArr3

{SumArr3 5.0.3}

```
int SumArr3(const bool pnArray[][jSize][kSize], const int nLength, const int mLength, const int lLength)
{
    int val =0;
    for (int i=0; i<nLength; i++){
        for (int j=0; j<mLength; j++) {
            for (int k=0; k<lLength; k++) {
                val += pnArray[i][j][k];
            }
        }
    }
    return val;
}
```

Used in section 5

5.0.4. WithinCompartment

{WithinCompartment 5.0.4}

```
int WithinCompartment( int i, int j, int k)
{
  return (0 <= i) && (i < iSize ) &&
         (0 <= j) && (i < jSize ) &&
         (0 <= k) && (i < kSize )     ;
}
```

Used in section 5

5.1. Conversion between cell index and coordinates.

The following functions convert between the index of a cell and its coordinates.

5.1.1. main program inits

This initialises the mapping between multidimensional array representation of the compartment and the one-dim index representation.

{main program inits 5.1.1}

```
    MatMappingInitialization(MapVM);
```

Used in section 2.2.1

5.1.1.1. MatMappingInitialization

We initialise the 3D `MapVW` array to hold the index of every cell.

{MatMappingInitialization 5.1.1.1}

```
void MatMappingInitialization(int (&MapVM)[iSize][jSize][kSize]){

    int index=0;
    for (int i=0; i<iSize; i++) {
        for (int j=0; j<jSize; j++) {
            for (int k=0; k<kSize; k++) {
                MapVM[i][j][k] = index;
                index++;
            }
        }
    }
}
```

Used in section 5

5.1.1.2. MatToVet

To get the index of a cell we simply return that entry.

{MatToVet 5.1.1.2}

```
int MatToVet(const int ik, const int jk, const int kk)
//This one is now obsolete: I can directly call the MapVM with the desired index.
{
  return MapVM[ik][jk][kk];
}
```

Used in section 5

5.1.2. VetToMat

To get the coordinates of a certain index we loop over all of `VetToMat` to find the entry that is equal to the one needed.

{VetToMat 5.1.2}

```
int VetToMat(const int valueFromVector, const int ZeroOneorTwo)
{
    int m=0;
    for (int i=0; i<iSize; i++) {
        for (int j=0; j<jSize; j++) {
            for (int k=0; k<kSize; k++) {
              if ( MapVM[i][j][k] == valueFromVector ) {

                if ( ZeroOneorTwo == 0 ) {
                  m=i;
                } else if (ZeroOneorTwo==1) {
                  m=j;
                } else {
                  m=k;
                }
              }
            }
        }
    }

    return m;
 }
```

Used in section 5

6. Fitness and replacement

6.1. Choose *reproducer* according to fitness

Choosing the reproducing cell is done in the function `ChooseAnElement`.

{Choose *reproducer* according to fitness 6.1}

```
int reproducer = ChooseAnElement( MatrixState, R);
```

Used in section 2.1.2

6.1.1. ChooseAnElement

This function calculates the fitness of each cell in the compartment, and then choses a cell to reproduce by fitness.

{ChooseAnElement 6.1.1}

```
int ChooseAnElement(const bool StateArray[iSize][jSize][kSize], const int R)
{
  {ChooseAnElement: initialise fitness array (MatrixFitness), 6.1.1.1}
  {Calculate fitness, 6.1.1.2}
  {Choose random cell, 6.1.1.9}
}
```

Used in section 5

6.1.1.1. ChooseAnElement: initialise fitness array (MatrixFitness)

{ChooseAnElement: initialise fitness array (MatrixFitness) 6.1.1.1}

```
    //*************** Initialise Fitness Array ********************************
    double MatrixFitness[iSize][jSize][kSize] = {{{ 0 }}};
```

Used in section 6.1.1

6.1.1.2. Calculate fitness

There are two separate models to calculate fitness

{Calculate fitness 6.1.1.2}

```
#ifdef CUMULATIVE
  {Calculate fitness cumulative, 6.1.1.3}
#else
  {Calculate fitness non cumulative, 6.1.1.4}
#endif
```

Used in section 6.1.1

6.1.1.3. Calculate fitness cumulative

In the cumulative model, with n neighbours that are of type 1, the fitness of a type 1 cell is $1 + nb - c$, and of a type 0 cell $1 + nb$. Thus we calculate fitness in the following way:

{Calculate fitness cumulative 6.1.1.3}

```
{set fitness to 1, 6.1.1.5}
{for every cell of type 1 add b to all neighbours, 6.1.1.6}
{subtract c from every cell of type 1, 6.1.1.8}
```

Used in section 6.1.1.2

6.1.1.4. Calculate fitness non cumulative

In the cumulative model, the difference is simply that if there is any neighbour of type 1, benefit is b, otherwise 0.

{Calculate fitness non cumulative 6.1.1.4}

```
{set fitness to 1, 6.1.1.5}
{for every cell of type 1 set all neighbours to 1+b, 6.1.1.7}
{subtract c from every cell of type 1, 6.1.1.8}
```

Used in section 6.1.1.2

6.1.1.5. set fitness to 1

{set fitness to 1 6.1.1.5}

```
    for (int i=0; i<iSize; i++){
        for (int j=0; j<jSize; j++) {
            for (int k=0; k<kSize; k++) {
                MatrixFitness[i][j][k] = 1;
            }
        }
    }
```

Used in sections 6.1.1.3 and 6.1.1.4

6.1.1.6. for every cell of type 1 add b to all neighbours

{for every cell of type 1 add b to all neighbours 6.1.1.6}

```
for (int i=0; i<iSize; i++) {
  for (int j=0; j<jSize; j++) {
    for (int k=0; k<kSize; k++) {             // loop over compartment
      if (StateArray[i][j][k] == 1 ) {        // if cell is mutant
        for (int m = -R; m <= R ; m++) {
          for (int l = -R ; l <= R ; l++) {
            for (int n= -R; n <= R ; n++) {   // loop over R distance neighbours
              if (  WithinCompartment( i+m, j+l, k+n) )
                {
                  MatrixFitness[i+m][j+l][k+n] += b ; // was * StateArray[i][j][k], but we know it is 1.
                }
            }
          }
        }
      }
    }
  }
 }
```

Used in section 6.1.1.3

6.1.1.7. for every cell of type 1 set all neighbours to 1+b

{for every cell of type 1 set all neighbours to 1+b 6.1.1.7}

```
for (int i=0; i<iSize; i++) {
  for (int j=0; j<jSize; j++) {
    for (int k=0; k<kSize; k++) {
      if ( StateArray[i][j][k] == 1 ) {
        for (int m = -R; m <= R ; m++) {
          for (int l = -R ; l <= R ; l++) {
            for (int n = -R; n <= R; n++) {
              if ( WithinCompartment(i+m, j+l, k+n) )
                {
                  MatrixFitness[i+m][j+l][k+n] = 1+b; // This was b, but I think it should be 1+b
                }
            }
          }
        }
      }
    }
  }
 }
```

Used in section 6.1.1.4

6.1.1.8. subtract c from every cell of type 1

{subtract c from every cell of type 1 6.1.1.8}

```
 for (int i=0; i<iSize; i++){
        for (int j=0; j<jSize; j++) {
            for (int k=0; k<kSize; k++) {
                if (StateArray[i][j][k]==1) {
                    MatrixFitness[i][j][k] += (-c) ; // was * StateArray[i][j][k], but we know it is 1.
                }
            }
        }
    }
```

Used in sections 6.1.1.3 and 6.1.1.4

6.1.1.9. Choose random cell

First, calculate non-normalised CDF for the fitness of all cells in compartment. Then sample 1 of them using this CDF.

{Choose random cell 6.1.1.9}

```
double FitnessArray[PopSize] = {0};
{transform fitness matrix to fitness array, 6.1.1.9.1}
{calculate (non normalised) CDF of fitness array, 6.1.1.9.2}
{sample 1 random cell using CDF, 6.1.1.9.3}
```

Used in section 6.1.1

6.1.1.9.1. transform fitness matrix to fitness array

{transform fitness matrix to fitness array 6.1.1.9.1}

```
//**************** Transforming Matrix Fitness to Array Fitness*************
int counter=0;
for (int i=0; i<iSize; i++) {
    for (int j=0; j<jSize; j++) {
        for (int k=0; k<kSize; k++) {
            FitnessArray [counter] = MatrixFitness[i][j][k];
            counter++;
        }

    }
}
```

Used in section 6.1.1.9

6.1.1.9.2. calculate (non normalised) CDF of fitness array

{calculate (non normalised) CDF of fitness array 6.1.1.9.2}

```
//******************** Cumulative vector **************************
double FitnessCDF[PopSize]={0};
for (int i=0; i<PopSize; i++){ FitnessCDF[i] =  FitnessArray[i];}
for (int i=1; i<PopSize; i++){ FitnessCDF[i] += FitnessCDF[i-1];}
```

Used in section 6.1.1.9

6.1.1.9.3. sample 1 random cell using CDF

{sample 1 random cell using CDF 6.1.1.9.3}

```
//******************** Return an Element weighted sampled **************************
return rnd::sample_1( FitnessCDF, PopSize);
```

Used in section 6.1.1.9

`rnd` is defined in `random.h`

6.1.1.9.4. includes +=

{includes 3} +=

```
#include "random.h" // for rnd::sample_1
```

Added to in sections 3.2.4.0.1, 5.0.1.0.1, 6.3.1.1.1, 6.3.1.2.1 and 6.3.1.4.1

Used in section 2.2.1

6.2. Choose random cell within range L *to be replaced*

{Choose random cell within range L *to be replaced* 6.2}

```
int to_be_replaced = NeighbourWithinL( reproducer ) ;
```

Used in section 2.1.2

6.2.1. NeighbourWithinL

Find a neighbour in radius L from focal, return its index. This is done by choosing random neighbour, and checking if it fulfils some conditions.

{NeighbourWithinL 6.2.1}

```
int NeighbourWithinL(const int addresoffocal)
{
    int a[3]={0,0,0};
    int focal[3] = { VetToMat(addresoffocal,0),
                     VetToMat(addresoffocal,1),
                     VetToMat(addresoffocal,0) } ;

    do{
        a[0] = focal[0] - L + IntegerRandom(2*L+1);
        a[1] = focal[1] - L + IntegerRandom(2*L+1);
        a[2] = focal[2] - L + IntegerRandom(2*L+1);
    } while ( ( (a[0] == focal[0]) &&   // continue while:
                (a[1] == focal[1]) &&
                (a[2] == focal[2])    ) // same location as focal
              ||
              ! WithinCompartment( a[0], a[1], a[2] ) ) ; // not in compartment

    return MatToVet(a[0], a[1], a[2]);
}
```

Used in section 5

6.3. Find cell of the same type as *to be replaced*, having maximum neigbours of same type as the *reproducer*, and replace it instead

{Find cell of the same type as *to be replaced*, having maximum neigbours of same type as the *reproducer*, and replace it instead 6.3}

```
Replacement( MatrixState, reproducer, to_be_replaced);
```

Used in section 2.1.2

6.3.1. Replacement

{Replacement 6.3.1}

```
void Replacement( bool (&MatrixSt)[iSize][jSize][kSize],
                  const int focal_Vet, const int replace_Vet)
{
  //    call_count++; I moved this out

    // If the focal and replace are different, do the replacement
  int focal[3] = { VetToMat( focal_Vet  , 0),
                   VetToMat( focal_Vet  , 1),
                   VetToMat( focal_Vet  , 2) } ;
  int replace[3] = { VetToMat( replace_Vet, 0),
                     VetToMat( replace_Vet, 1),
                     VetToMat( replace_Vet, 2) } ;
  bool focal_type =   (MatrixSt)[ focal[0]   ][ focal[1]   ][ focal[2] ] ;
  bool replace_type = (MatrixSt)[ replace[0] ][ replace[1] ][ replace[2] ] ;

  if( focal_type != replace_type) {
        if ( focal_type == 1) {

          {Calculate number of immediate neighbours of type 1 for each cell of type 0, 6.3.1.1}
          {Find the cells with the maximal number of neighbours of type 1, 6.3.1.3}

           // choose one of them, those with the maximal #of neighbours diff from them.
           int choo= VectorAddresMaximum[ IntegerRandom(static_cast<int>(VectorAddresMaximum.size()))];

           // Finally, set that one to 1, we replicated.
           MatrixSt[VetToMat(choo, 0)][VetToMat(choo, 1)][VetToMat(choo, 2)]= 1 ;

           // Erase the vectors.
           VectorZeroAddress.erase (VectorZeroAddress.begin(), VectorZeroAddress.begin()+VectorZeroAddress.size());
           VectorNOnes.erase (VectorNOnes.begin(), VectorNOnes.begin()+VectorNOnes.size());
           VectorAddresMaximum.erase (VectorAddresMaximum.begin(),
VectorAddresMaximum.begin()+VectorAddresMaximum.size());
        } else { // if the focal isn't 1, i.e. if the focal is 0.
          {Calculate number of immediate neighbours of type 0 for each cell of type 1, 6.3.1.2}
          {Find the cells with the maximal number of neighbours of type 0, 6.3.1.4}

          // chose a random maximal cell
          int choo=VectorAddresMaximum[IntegerRandom(static_cast<int>(VectorAddresMaximum.size()))];

          // set it to 0, i.e. to the value of the focal
            MatrixSt[VetToMat(choo, 0)][VetToMat(choo, 1)][VetToMat(choo, 2)]= 0 ;


            VectorOneAddress.erase (VectorOneAddress.begin(), VectorOneAddress.begin()+VectorOneAddress.size());
            VectorNZeros.erase (VectorNZeros.begin(), VectorNZeros.begin()+VectorNZeros.size());
            VectorAddresMaximum.erase (VectorAddresMaximum.begin(),
VectorAddresMaximum.begin()+VectorAddresMaximum.size());




        }

    }

}
```

Used in section 5

6.3.1.1. Calculate number of immediate neighbours of type 1 for each cell of type 0

`counter2` records the number of neighbours of type 1. `VectorZeroAddress` records location of each cell that has more than one neighbour of type 1. `VectorNOnes` records the number of such neighbours.

{Calculate number of immediate neighbours of type 1 for each cell of type 0 6.3.1.1}

```
vector<int> VectorZeroAddress;
vector<int> VectorNOnes;

// Find places in MatrixSt that are 0 and have
// neighbours that are 1.
// Store the place and how many neighbours are 1 in VectorNOnes.
// neighbourhood is -1, 0, +1.
for (int i=0; i<iSize; i++) {
  for (int j=0; j<jSize; j++) {
    for (int k=0; k<kSize; k++){
      if (MatrixSt[i][j][k] == 0 ) { // cell is 0
        int counter2=0;
        for (int ik= i-1; ik<= i+1; ik++) {
          for (int jk= j-1; jk<= j+1; jk++) {
            for(int kk= k-1; kk <= k+1; kk++){
              if( WithinCompartment(ik, jk, kk) &&
                  MatrixSt[ik][jk][kk] == 1         )
                counter2++;
            } // for kk
          } // for jk
        } // for ik
        if (counter2 > 0) {
          VectorZeroAddress.push_back( MatToVet( i, j, k) ); // record address of 0 cell
          VectorNOnes.push_back( counter2 ); // record number of 1
        }
      } // if MatrixSt
    } // for k
  } // for j
 } // for i
//############################
```

Used in section 6.3.1

Since `VectorNOnes` and `VectorZeroAddress` are `vector<int>`, we need

6.3.1.1.1. includes +=

{includes 3} +=

```
#include <vector>
```

Added to in sections 3.2.4.0.1, 5.0.1.0.1, 6.1.1.9.4, 6.3.1.2.1 and 6.3.1.4.1

Used in section 2.2.1

6.3.1.2. Calculate number of immediate neighbours of type 0 for each cell of type 1

`counter2` records the number of neighbours of type 0. `VectorZeroAddress` records location of each cell that has more than one neighbour of type 0. `VectorNOnes` records the number of such neighbours.

{Calculate number of immediate neighbours of type 0 for each cell of type 1 6.3.1.2}

```
vector<int> VectorOneAddress;
vector<int> VectorNZeros;

// Find places in MatrixSt that are 1 and have
// neighbours that are 0.
// Store the place and how many neighbours are 0 in VectorNZeros.
// neighbourhood is -1, 0, +1.

for (int i=0; i<iSize; i++) {
  for (int j=0; j<jSize; j++) {
    for (int k=0; k<kSize; k++){
      if( MatrixSt[i][j][k]==1 ) { // cell is 1
        int counter2=0;
        for (int ik=-1+i; ik<2+i; ik++) {
          for (int jk=-1+j; jk<2+j; jk++) {
            for(int kk=-1+k; kk<2+k; kk++){
              if ( WithinCompartment(ik, jk, kk) &&
                   MatrixSt[ik][jk][kk] == 0        )
                counter2++;
            }
          }
        }
        if (counter2 !=0) {
          VectorOneAddress.push_back( MatToVet( i, j, k)); // record address of 1 cell
          VectorNZeros.push_back( counter2 ); // record number of 0
        }
      }
    }
  }
 }
//##############################
```

Used in section 6.3.1

Since `VectorNZeros` and `VectorOneAddress` are `vector<int>`, we need

6.3.1.2.1. includes +=

{includes 3} +=

```
#include <vector>
```

Added to in sections 3.2.4.0.1, 5.0.1.0.1, 6.1.1.9.4, 6.3.1.1.1 and 6.3.1.4.1

Used in section 2.2.1

6.3.1.3. Find the cells with the maximal number of neighbours of type 1

{Find the cells with the maximal number of neighbours of type 1 6.3.1.3}

```
vector<int> VectorAddresMaximum;

// Among VectorNOnes, find those that are equal to the maximum,
// and put those on VectorAddresMaximum.
// Notice that max_element is computed every step of the loop,
// though it could be computed just once.
for (unsigned int i=0; i < (VectorZeroAddress.size()); i++) {
  if ( VectorNOnes[i] == *max_element( begin( VectorNOnes), end( VectorNOnes) ) ) {
    VectorAddresMaximum.push_back( VectorZeroAddress[ i]);
  }
}
```

Used in section 6.3.1

6.3.1.4. Find the cells with the maximal number of neighbours of type 0

{Find the cells with the maximal number of neighbours of type 0 6.3.1.4}

```
vector<int> VectorAddresMaximum;

// Among VectorNZeros, find those that are equal to the maximum,
// and put those on VectorAddresMaximum.
// Notice that max_element is computed every step of the loop,
// though it could be computed just once.

for (unsigned int i=0; i< (VectorOneAddress.size()); i++) {
  if( VectorNZeros[i] == *max_element( begin(VectorNZeros), end(VectorNZeros) )) {
    VectorAddresMaximum.push_back( VectorOneAddress[ i] );
  }
}
```

Used in section 6.3.1

Since `VectorAddresMaximum`is `vector<int>`, we need `<vector>`, and for `max_element` we need `<algorithm>`

6.3.1.4.1. includes +=

{includes 3} +=

```
#include <vector>
#include <algorithm>
```

Added to in sections 3.2.4.0.1, 5.0.1.0.1, 6.1.1.9.4, 6.3.1.1.1 and 6.3.1.2.1

Used in section 2.2.1
